# Nucleotide modifications within bacterial messenger RNAs regulate their translation and are able to rewire the genetic code

**DOI:** 10.1093/nar/gkv1182

**Published:** 2015-11-17

**Authors:** Thomas Philipp Hoernes, Nina Clementi, Klaus Faserl, Heidelinde Glasner, Kathrin Breuker, Herbert Lindner, Alexander Hüttenhofer, Matthias David Erlacher

**Affiliations:** 1 Division of Genomics and RNomics, Biocenter, Medical University of Innsbruck, 6020 Innsbruck, Austria; 2 Division of Clinical Biochemistry, Biocenter, Medical University of Innsbruck, 6020 Innsbruck, Austria; 3 Institute of Organic Chemistry and Center for Molecular Biosciences (CMBI), University of Innsbruck, 6020 Innsbruck, Austria

## Abstract

Nucleotide modifications within RNA transcripts are found in every organism in all three domains of life. 6-methyladeonsine (m^6^A), 5-methylcytosine (m^5^C) and pseudouridine (Ψ) are highly abundant nucleotide modifications in coding sequences of eukaryal mRNAs, while m^5^C and m^6^A modifications have also been discovered in archaeal and bacterial mRNAs. Employing *in vitro* translation assays, we systematically investigated the influence of nucleotide modifications on translation. We introduced m^5^C, m^6^A, Ψ or 2′-O-methylated nucleotides at each of the three positions within a codon of the bacterial ErmCL mRNA and analyzed their influence on translation. Depending on the respective nucleotide modification, as well as its position within a codon, protein synthesis remained either unaffected or was prematurely terminated at the modification site, resulting in reduced amounts of the full-length peptide. In the latter case, toeprint analysis of ribosomal complexes was consistent with stalling of translation at the modified codon. When multiple nucleotide modifications were introduced within one codon, an additive inhibitory effect on translation was observed. We also identified the m^5^C modification to alter the amino acid identity of the corresponding codon, when positioned at the second codon position. Our results suggest a novel mode of gene regulation by nucleotide modifications in bacterial mRNAs.

## INTRODUCTION

Modifications within RNA transcripts are highly abundant and found to be essential for numerous biological processes (1). More than 140 different modification types in all RNA species have been identified (2). Thereby, the largest number and diversity of nucleotide modifications have been found in transfer RNAs (tRNAs) (3) which have been reported to be essential for efficient and accurate translation (4,5).

**Figure 1.**
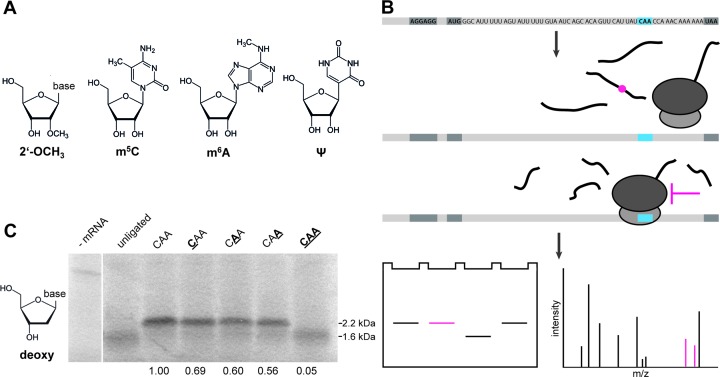
Overview of the experimental setup. (**A**) The chemical structures of investigated nucleotide derivatives are depicted. Nucleotides carrying methylations either at the ribose (2′-OCH_3_) or the base (5-methylcytosine (m^5^C) and N^6^-methyladenosine (m^6^A)) as well as pseudouridines (Ψ) were site-specifically introduced into mRNAs and translated. (**B**) mRNAs carrying a modified codon (blue) were translated *in vitro*. The produced peptides were analyzed by SDS-PAGE, for determination of protein amounts and size, and by mass spectrometry for their amino acid composition; this allows the elucidation of the ratio of full length (2.2 kDa) versus truncated peptides (1.6 kDa; black lines) and detection of altered amino acid sequences (red line). (**C**) SDS-PAGE of translation products of unmodified mRNAs and mRNAs carrying deoxy-nucleotides at the CAA codon at the first, second or third codon position (bold and underlined). Translation in of the unligated 5′-part as well as reactions in the absence of mRNAs were used as negative controls.

Ribosomal RNAs (rRNAs) mainly harbor pseudouridines (Ψ) or 2′-O-methylated nucleotides (Figure 1A), which accumulate in functional and structurally conserved regions (6). Thereby, they are involved in numerous aspects of ribosome assembly and translation. Nevertheless, until now the precise functional roles of these modifications as well as their impact on translation remain unsolved (7–9).

More than 10 ‘non-standard’ nucleotides have been described in eukaryal mRNAs from which the 5′-cap modification and variants of the 5′-cap are the best known examples (10). N^6^-methyladenosine (m^6^A) and, more recently, 5-methylcytosine (m^5^C) and pseudouridine (Figure 1A) were reported to be present within 5′- and 3′-untranslated regions (UTRs) as well as within coding sequences of mRNAs (11–16). In particular, m^6^A represents the most abundant modification within eukaryal mRNAs (11). This methylation is found in eukaryal mRNA species from yeast to mammals. Thereby, m^6^A modifications are located in highly conserved mRNA regions and also appear to be introduced into mRNAs dependent on the cell type (reviewed in 17,18). These m^6^A modifications are enriched in the vicinity of stop codons, potentially influencing mRNA stability or its regulation through miRNAs (19). Interestingly, m^6^A has been found to be enzymatically eliminated from mRNAs, implicating m^6^A to be a dynamic regulator of gene expression (20). Very recently, also bacterial mRNAs have been reported to harbor numerous m^6^A nucleotides within their coding sequences, emphasizing the importance of m^6^A not only in eukaryotes (21).

m^5^C is described to be highly abundant in eukaryal mRNAs. Through transcriptome analysis, more than 10 000 m^5^C sites have been identified in mRNAs (12). This modification was also reported to be present in Archaea (22), but lacks its identification in bacterial mRNAs, up till now.

In 2014, Ψ has been described in coding sequences of mRNAs (13–16). In these reports, several hundred Ψs were identified in yeast and in human mRNAs through transcriptome-wide screens. Whereas Ψs were found to influence the half-life of mRNAs (23) or alter the thermodynamic stability of RNA structures (24,25), their direct impact on translation remains unknown. It has been speculated that Ψs, located within open reading frames of mRNAs, might cause an amino acid substitution during translation (13,26). Ψs, located within stop codons, resulted in nonsense suppression and thus bypassed translation termination by incorporation of specific amino acids in *Escherichia coli* (27,28). According to computational calculations, Ψ located in sense codons might alter the codon identity, thus facilitating altered codon recognition (29).

The 2′-O-methylation (2′-OCH_3_) of riboses might also be employed for translation regulation. So far, this modification has not unambiguously been identified within coding sequences of mRNAs. However, distinct RNA-protein complexes are present within eukaryal cells, which might introduce this modification into open reading frames of mRNAs. The insertion of 2′-O-methylations into rRNAs and snRNAs is catalyzed by an RNA guided ribonucleo–protein complex, designated as C/D box small nucleolar RNP complex (30). In addition, so-called ‘orphan’ snoRNAs are predicted to target other RNA species than ribosomal RNAs or snRNAs (31). In particular, it has been suggested that the orphan snoRNA SNORD-115 may guide 2′-O-methylation of the 5-HT_2C_ pre-mRNA, thereby regulating gene expression (32,33).

To elucidate the roles of mRNA modifications in protein synthesis, several studies were carried out employing various translation systems and strategies to introduce nucleotide modifications into mRNAs (27–28,34–35). However, the results of these studies are contradictory and the effects could not univocally be attributed to either the translation process *per se* or to indirect effects such as altered mRNA stability or mRNA-protein interactions changing translation efficiency.

Hence, in this study we aimed to get a better understanding of the impact of single nucleotide modifications on protein synthesis in bacterial mRNAs. The modified RNA nucleotides were specifically introduced at defined codon positions (i.e. first, second or third position, respectively) within the bacterial ErmCL mRNA. Employing an *E. coli* based translation system we examined the impact of the modified codons on protein synthesis. In addition, we characterized translation products by mass spectrometry to investigate whether mRNA modifications within codons would alter insertion of the cognate amino acid, thus ‘rewiring’ the genetic code. For protein characterization, we applied a liquid chromatography-mass spectrometry (LC-MS) coupling. This highly sensitive method enables detection of peptides present in minute amounts, in addition to their precise amino acid composition.

Our results demonstrate, that indeed mRNA modifications are able to strongly regulate protein synthesis and thus might add another layer of regulation to the complex mechanism of gene expression.

## MATERIALS AND METHODS

### Constructs

ErmCL nucleotide sequence was cloned into the BamHI/EcoRI sites of pUC19. 5′ of the mRNA sequence a T7 promotor, an enhancer sequence and a Shine Dalgarno sequence were introduced allowing an efficient translation (Supplementary Figure S1A). All ErmCL variants (Q15P, Q15K, Q15F) and ErmCL fragments for splinted RNA ligations were amplified by polymerase chain reaction (PCR) from pUC19-T7-ErmCL.

### Transcription

Templates for transcription were generated by PCR from pUC19-T7-ErmCL or by EcoRI linearization of pUC19-T7-ErmCL for subsequent run off transcription. The templates were purified via the QIAquick PCR purification kit or phenol-chloroform extraction. The transcripts were generated according to the manual using the RiboMAX Large Scale RNA Production System (Promega) or the HiScribe T7 Quick High Yield RNA Synthesis kit (New England Biolabs). Subsequently, the mRNAs were purified via illustra MicroSpin G-25 columns (GE Healthcare) and phenol-chloroform extraction. Whenever needed transcripts were gel purified employing 8% polyacrylamid gels as described below.

### Splinted RNA ligation

Splinted ligations of the RNA oligonucleotides were generally performed as described (36). RNA oligonucleotides with a 5′ phosphorylation were chemically synthesized by IDT or Dharmacon. In a standard preparation, 60 pmol 5′ transcript were mixed with 90 pmol of the synthesized RNA oligonucleotide 5′-P-AUUAUCAACCAAACAAAAAAUAA-3′ (modified codon underlined) and 60 pmol of the DNA splint 5′-TTTGTTTGGTTGATAATGAACTGTG-3′ in a volume of 16.2 μl. The reaction was incubated at 94°C for 3 min and subsequently cooled down to room temperature over 15 min. The enzymatic RNA ligation was performed by either T4 DNA ligase (Fermentas) or RNA ligase 2 (New England Biolabs). The reaction was supplemented with 2.4 μl 10× ligation buffer (Fermentas/New England Biolabs), 2.4 μl PEG 4000 (Fermentas), 40U RiboLock RNase Inhibitor (Thermo Scientific) and 10U T4 DNA ligase/RNA ligase 2 to a final volume of 24 μl and incubated at 35–37°C for 4 h. Subsequently, DNA splints were digested by 10U DNase I (Fermentas) at 37°C for 30 min. The reaction was purified employing phenol–chloroform extraction, precipitated and the RNAs were separated on an 8% polyacrylamide gel. The ligated mRNAs were cut out from the gel and were passively eluted overnight at 4°C into gel elution buffer (300 mM NaCl, 0.2% sodium dodecyl sulphate, 60 mM NaOAc pH 5.2). The eluate was precipitated and the mRNAs dissolved in ddH_2_O.

### *In vitro* translation

70S ribosomes and S100 extracts were prepared as previously described (37). The i*n vitro* translation reaction employing the *E. coli* whole cell extracts was performed as reported (38). For cell free *in vitro* translation the PURExpress Δ Ribosome Kit (New England Biolabs) was employed. In a typical reaction, 6–10 pmol mRNA were mixed with 3 μl of the manufacturer's solution A and 0.9 μl factor mix. Several concentrations of 70S ribosomes were tested ranging from 5 to 18 pmol, without any significant differences. For the described experiments 5 pmol of ribosomes were employed for one reaction. Between 5 and 10 μCi of [^35^S]Met/[^35^S]Cys were added to the translation mix. The reactions were incubated for the indicated period of time at 37°C and subsequently separated on Novex 16% Tris-Tricine gels (Life technologies) (39). The gel was exposed to a phosphorimager screen and scanned using a STORM 840 scanner.

For mass spectrometry ErmCL peptides were translated utilizing the PURExpress translation system and purified employing Vivaspin 2 (5 kDa, Hydrosart) centrifugal concentrators (Sartorius). The reactions were adjusted to 70% acetonitrile and 0.1% formic acid to prevent binding of the peptide to the membrane. The purification was performed as described by the manufacturer. Subsequently, the purified peptides were acetone precipitated and resuspended in ddH_2_O for mass spectrometry.

### Mass spectrometry

Purified ErmCL peptides were analyzed using a Dionex, UltiMate 3000 nano-HPLC system (Germering, Germany) coupled via nanospray ionization source to a Thermo Scientific Q Exactive Plus mass spectrometer (Vienna, Austria). Peptides were separated on a fritless fused-silica column (75 μm i.d. × 280 μm o.d. × 10 cm length) packed with 3 μm reversed-phase C18 material (Reprosil). Solvents for HPLC were 0.1% formic acid (solvent A) and 0.1% formic acid in 85% acetonitrile (solvent B). The gradient started at 4% B. The concentration of solvent B was increased linearly from 4 to 50% during 50 min and from 50 to 100% during 5 min. A flowrate of 250 nl/min was applied.

The Q Exactive Plus mass spectrometer was operating in data dependent mode to switch between MS and MS/MS acquisition. Full scan MS spectra were acquired with a resolution of R = 70 000. Up to 12 of the most intense ions detected in the full scan MS were sequentially isolated and fragmented using higher energy collision dissociation (HCD) applying a normalized collision energy of 28.0. Fragments were scanned with a resolution of *R* = 35 000.

Database search was performed using ProteomeDiscoverer (Version 1.4, Thermo Scientific) with search engine Sequest HT. MS/MS spectra were searched against an *E. coli* database (Uniprot, strain K12, last modified April 2015, 4305 entries) to which 21 different ErmCL peptides sequences were added. The following settings were applied: the b and y ions were used for spectrum matching and scoring; precursor mass tolerance was set to 10 ppm; fragment mass tolerance was 0.05 Da; false discovery rate (FDR) was set to 0.01 (1%); and N-terminal protein formylation was used as variable modification.

### Toeprint

*In vitro* translation of ErmCL-CAT mRNA was performed with the cell-free *in vitro* translation kit PUREexpress Δ RF123 (New England Biolabs) in a total volume of 6.25 μl. For the positive control the 22nd codon of the ErmCL-CAT mRNA was mutated to the stop codon UAA, and the release factors were omitted from the translation reaction. Toeprinting was essentially performed as described (40) with the following changes. The *in vitro* translation was carried out for 15 min at 37°C and the final toeprinting volume was 10.1 μl. The DNA primer (5′-TTAGTGTAGAAACTGCCGG-3′) complementary to the 3′-end of the ErmCL-CAT mRNA was used. After ethanol precipitation the pellets were resuspended in equal amounts of formamide RNA loading dye and separated on a denaturing 8% polyacrylamide gel, exposed on a phosphorimager screen and analyzed on the STORM 840 scanner. Sequencing lanes were performed as described (41).

### Leucine/isoleucine incorporation

To monitor leucine misincorporation, [^3^H]-Leu was provided to the PURExpress translation, the corresponding Tris-tricine gels were bathed in EN^3^HANCE autoradiography enhancer solution (PerkinElmer) as recommended by the manufacturer and exposed to BioMax MS films (Kodak).

For determination of isoleucine incorporation, [^14^C] labeled isoleucine was added to the PURExpress translation reaction. All naturally occurring isoleucine codons were exchanged to methionine codons to allow the determination of putative isoleucine incorporation at the modified codon. Subsequent to Tris-tricine gel electrophoresis, gels were dried and exposed to a phosphoimager screen and scanned, employing a STORM 840 scanner.

## RESULTS

### Experimental setup

The present study aimed to identify the influence of single nucleotide modifications, located within coding sequences of mRNAs, on the translation machinery. Thereby, we directly correlated the presence of nucleotide modifications on peptide synthesis in respect to peptide amounts and amino acid compositions. To that end, we introduced modifications at defined positions within codons of bacterial mRNAs (Supplementary Figure S1).

The site-specific incorporation of nucleotide modifications into mRNAs is based on a splinted ligation setup (36,42). Thereby, the 5′-RNA part of the mRNA was generated by T7 *in vitro* transcription. The 3′-half, carrying the modified nucleotide, was chemically synthesized thus ensuring a defined position of the nucleotide derivative (Supplementary Figure S1). Subsequently, the ligation product was gel-purified and used for *in vitro* translation. This setup assured that every mRNA carried the identical modification at the same position, thus providing a homogenous mRNA population.

As a model system, we employed ErmCL mRNA (40) for two reasons: firstly, the length of the mRNA allowed an efficient separation of the ligated product (101 nts) from the unligated 5′- and 3′- halves (78 and 23 nts, respectively); secondly, the size of the encoded peptide permits distinguishing between the full-length (2.2 kDa) and the truncated product (1.6 kDa) by sodium dodecyl sulphate-polyacrylamide gel electrophoresis (SDS-PAGE) analysis (Figure 1B). Thereby, modifications were introduced within the 15th codon (CAA) of the ErmCL mRNA, encoding for glutamine. Whenever the experimental setting allowed maintaining the ErmCL wild-type (wt) sequence (e.g. for the incorporation of deoxy-nucleotides or 2′-O-methylations within riboses), the base sequence of the codon was not altered. For base modifications (i.e. to investigate the effects of Ψs at all three positions), the codon identity was changed, but the position of the modified codon within the ErmCL mRNA was kept at the identical site. In contrast to earlier studies, where mRNAs carried randomly inserted modified nucleotides (35,43), our approach allows a defined incorporation of single modified nucleotides and thus enables the investigation of the correlation between the modification and its influence on the translation machinery.

### Incorporation of deoxy-nucleotides

Previously, deoxy-nucleotide substitutions within codon/anticodon base pairs were reported to strongly inhibit distinct steps of protein synthesis in *E. coli* (44–46). To validate this finding by our *in vitro* translation approach, we generated mRNAs carrying a deoxy-nucleotide at either the first, second or third codon position. In addition, an mRNA carrying an all DNA-containing codon (i.e. deoxy-nucleotides at all three codon positions) was synthesized. Employing an *in vitro* translation system based on *E. coli* whole cell lysates (38), we translated the modified or unmodified ErmCL mRNA, respectively, and subsequently analyzed the resulting peptide products (Figure 1C). The unligated ErmCL transcript, harboring the 5′ half of the mRNA only, did not result in a full-length peptide (2.2 kDa), but showed a signal sized ∼1.6 kDa, consistent with translation of the truncated variant of the ErmCL mRNA (Figure 1C). In contrast, the ligated, full-length wt mRNA resulted in the synthesis of a full-length ErmCL peptide (Figure 1C).

The introduction of a single deoxy-nucleotide at any of the 3 codon-positions reduced the yield of the full-length peptide by 30–40%. In contrast, the ‘all DNA’ codon completely aborted peptide synthesis at the modified codon (Figure 1C). These results are in line with earlier studies, reporting a 50–75%-fold decrease at distinct steps of translation (e.g. A-site tRNA binding or EF-Tu GTPase activation, respectively) when introducing single deoxy-nucleotides; an almost complete translational inhibition at the modified codon was observed introducing two or more deoxy-nucleotides (45,46).

Interestingly, the effects of the deoxy mRNA modification appeared to be slightly more pronounced when only distinct steps of protein synthesis were examined compared to the final protein product, involving multiple steps of translation. This underlines the importance of investigating the effects of mRNA modifications not only at separate steps of translation but also in the context of entire synthesis of the respective protein.

Subsequent to validation of the experimental setup, we established a purification protocol for the ErmCL peptide for mass spectrometry analysis (Figure 1B). Mass spectrometry allows characterization of the peptide in respect to amino acid composition and, consequently, the identification of a potential rewiring of the genetic code by incorporation of non-cognate amino acids at the mutated codon (Figure 1B).

### m^6^A

**Figure 2.**
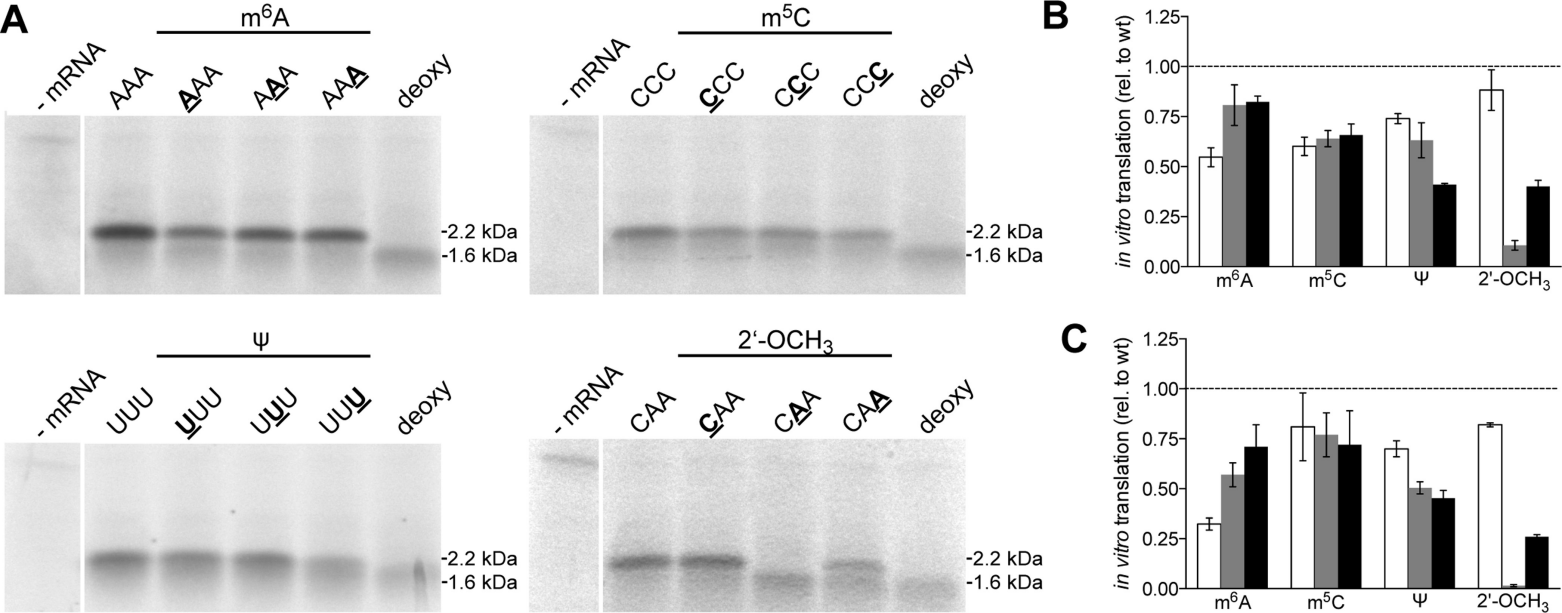
*In vitro* translation of ErmCL mRNAs carrying modified nucleotides at defined codon positions. (**A**) SDS-PAGE analysis of *in vitro* translated mRNA constructs, carrying the depicted modifications at the indicated positions (bold and underlined); mRNAs containing a codon, harboring three deoxy riboses at codon position 15, serve as controls (deoxy) (**B**) Quantification of at least three independent experiments of *in vitro* translated mRNAs harboring modified nucleotides at the first (white), second (gray) and third codon position (dark gray), respectively, employing the whole extract system or (**C**) the PURExpress system. The product yield of the translation of the unmodified mRNA (wt) was set to one. Values are depicted as mean ± SEM.

As a second modification, we introduced m^6^A at the 15th codon of the ErmCL mRNA (Figure 1A). m^6^A has been identified within eukaryal (47) as well as in bacterial mRNAs (21). The RNA sequence at the modified codon was changed from CAA to AAA encoding lysine while the sequence of the unmodified control mRNA was changed accordingly. m^6^A was either introduced at the first, second or third codon position, respectively, and the translation product was analyzed employing SDS-PAGE. Interestingly, m^6^A at the first nucleotide of the codon reduced translation of the corresponding full-length-peptide by about 50% (Figure 2A and B). At the remaining codon positions (i.e. two and three) about 20% reduction of the full-length protein product was observed in line with previous reports (48). As seen for deoxy nucleotides, incorporation of three consecutive m^6^A nucleotides within the lysine codon (AAA) completely aborted peptide synthesis (Supplementary Figure S4).

When employing whole protein extracts for protein synthesis, we cannot exclude the possibility that the respective mRNA modification does not only influence translation of the mRNA but in addition might influence its stability. To exclude this possibility, we radioactively labeled the modified and unmodified mRNAs and compared their stability during translation by PAGE; in these experiments, we did not observe any differences in the stabilities of modified versus unmodified mRNAs, respectively (data not shown).

In addition, mRNA modifications might also indirectly affect translation, e.g. by altered binding affinities of modified mRNAs to RNA binding proteins thus reducing or increasing expression efficiency (49). To exclude this possibility, we employed the PURExpress *in vitro* translation system to translate modified mRNAs (50). This system is based on a minimal number of purified and recombinant components of proteins required for translation.

Employing the PURE system, we confirmed the results from the cell free translation assay. However, the effects of mRNA modifications on translation were significantly more pronounced (Figure 2C and Table 1). Thereby, m^6^A at the first position resulted in a reduction of the full length 2.2 kDa translation product by about 75%, compared to 50% employing the whole cell lysate-based system. The inhibitory effect of the modifications at the second and third position was also more pronounced, showing ∼50% decrease in product yield (Figure 2C and Table 1). As observed for the whole protein extract, mRNA stability was unaffected by the modification in the PURExpress system (data not shown).

**Table 1.** Table depicting the effects of the modifications on translation reactions

Modification	Position	Rel. translation	Recoding
2′-OCH_3_	2	0.02 ± 0.01	-
2′-OCH_3_	3	0.26 ± 0.01	-
m^6^A	1	0.32 ± 0.03	-
Ψ	3	0.45 ± 0.04	-
Ψ	2	0.51 ± 0.03	-
m^6^A	2	0.57 ± 0.06	-
Ψ	1	0.70 ± 0.04	-
m^6^A	3	0.71 ± 0.11	-
m^5^C	3	0.72 ± 0.17	-
m^5^C	2	0.77 ± 0.11	+
m^5^C	1	0.81 ± 0.17	-
2′-OCH_3_	1	0.82 ± 0.01	-

The produced peptides employing modified mRNAs were related to translation products utilizing unmodified mRNAs (rel. translation). Modifications causing an amino acid substitution are indicated by +. Values are depicted as means ± SEM.

**Figure 3.**
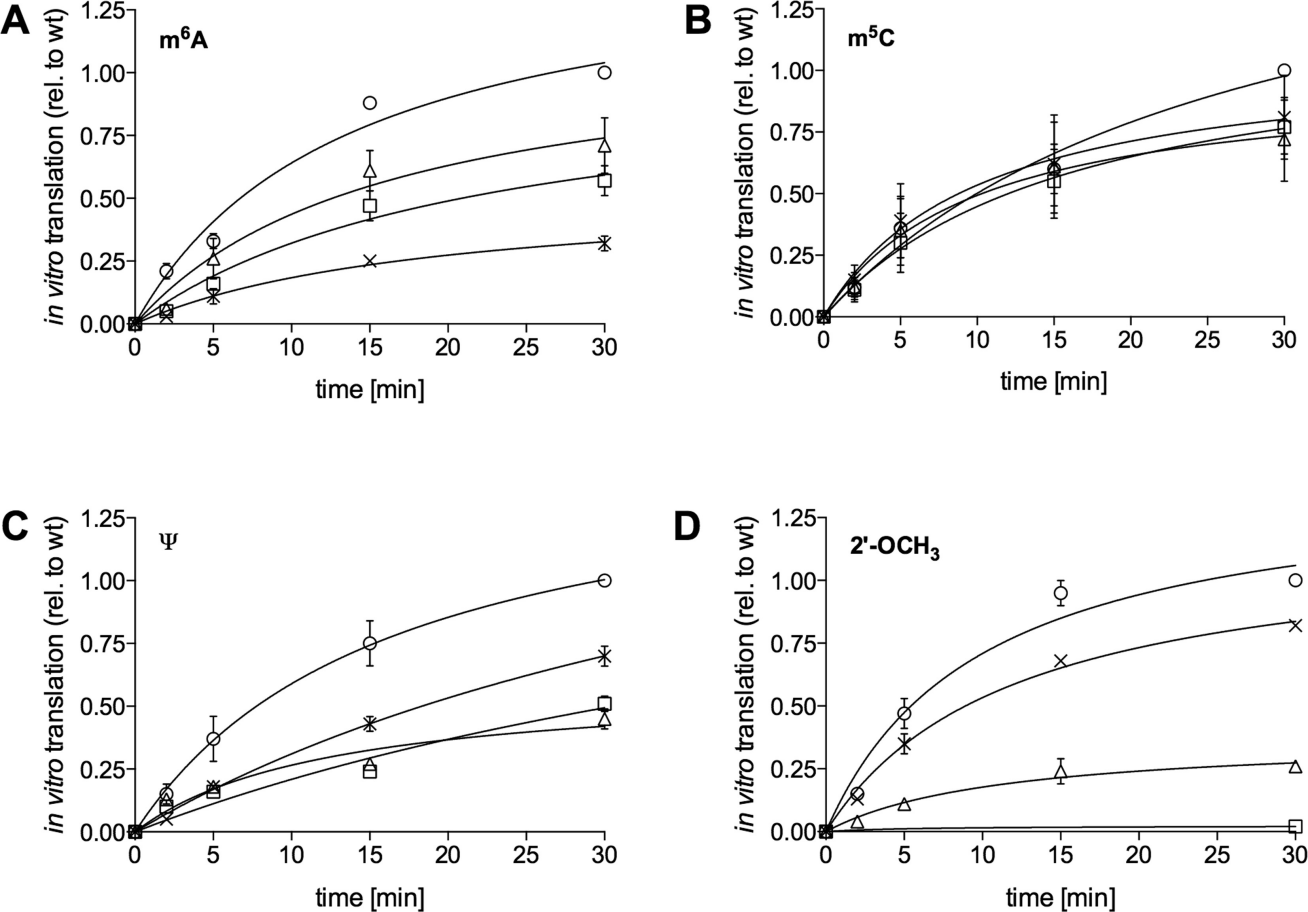
Time course experiments of the PURExpress system translating unmodified or modified mRNAs, respectively; translation products of the 30 min time point of unmodified mRNAs (wt) (o) were set to one. The nucleotide modifications (**A**) m^6^A, (**B**) m^5^C, (**C**) Ψ and (**D**) 2′-OCH_3_ were individually introduced at the first (X), the second (☐) or the third codon position (Δ), respectively. Each time point is represented by at least three independent measurements. The values are depicted as mean ± SEM.

We next investigated, whether premature translation termination would already be visible at earlier time points of reaction. Therefore, time course experiments were performed, employing the recombinant expression system (Figure 3). Thereby, we observed that indeed also at very early time points, the synthesis of the full-length peptide was significantly reduced due to the m^6^A-modified nucleotides (Figure 3A). In addition to determining the amounts of the respective peptides, also its amino acid composition was evaluated. To that end, peptides were purified employing centrifugal concentrators, followed by mass spectrometry (MS). Independent of the codon position of m^6^A within the ErmCL mRNA, we could only identify the wt ErmCL peptide sequence, thus excluding a rewiring of the genetic code by incorporation of non-cognate amino acids (Supplementary Figure S2A).

### m^5^C

To investigate the impact of m^5^C on translation, we altered the mRNA sequence at the 15th codon from CAA to CCC, encoding the amino acid proline. mRNAs carrying m^5^C at any of the three codon positions resulted in ∼40% decrease in product yield (Figure 2A and B). Also the utilization of the PURE translation system showed similar results (Figure 2C and Table 1). This was also reflected by the time course experiments (Figure 3B), excluding the possibility of time dependence.

**Figure 4.**
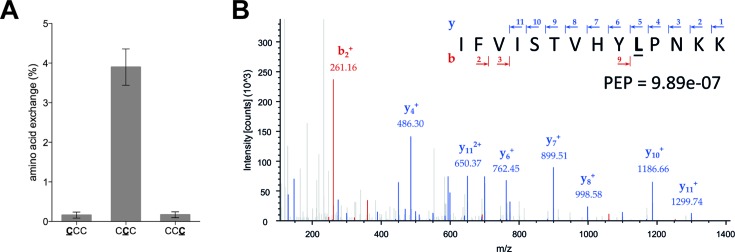
Translational recoding due to the presence of m^5^C. (**A**) Quantification of the peptides carrying an exchanged amino acid at the 15th codon (CCC) of ErmCL, encoding proline. Leucine (encoded by CUC) was only detected when m^5^C (bold and underlined) was present at the second codon position. The values are depicted as mean ± SEM. (**B**) MS/MS spectrum of the ErmCL peptide encoded by the mRNA harboring m^5^C at the second nucleotide of the 15th codon. Annotated peptide fragments are shown in blue (y-ions) and red (b-ions), according to the general nomenclature of fragment ions. The posterior error probability (PEP) indicates the probability that the annotation is incorrect.

The analysis of the peptide product by mass spectrometry did not reveal a change of the amino acid sequence, when introducing m^5^C at the first or third codon position, respectively. However, an amino acid change was detected by introducing m^5^C at the second nucleotide of the codon (Figure 4A and B). From mass spectrometry analysis it was estimated that about 4% of proline residues were substituted by either isoleucine or leucine (Figure 4A). Because the identity of the substituted amino acid cannot be determined by MS, we performed *in vitro* translation reactions in the presence of either [^3^H]-labeled leucine or [^14^C]-labeled isoleucine. Peptide products were analyzed by SDS-PAGE followed by autoradiography. Thereby, we detected the leucine but not isoleucine incorporation, instead of proline, into ErmCL peptides when m^5^C was introduced at the second codon position (Supplementary Figure S5). In contrast, the ErmCL peptide translated from the unmodified mRNA did not contain any detectable amounts of leucine. To exclude the possibility that this recoding event is a result of deamination of m^5^C resulting in m^5^U (5-methyluridine), we performed mass spectrometry analysis of the modified RNA. No indication for the presence of m^5^U could be found (Supplementary Figure S6).

### Ψ

Very recently, Ψs have been discovered to be present within the coding sequences of mRNAs (13–16). Hence, we investigated the influence of Ψs on translation and changed the sequence at the respective codon within the ErmCL mRNA to UUU encoding phenylalanine, thus providing the possibility to introduce Ψ within every position of the codon. Translation of mRNAs, carrying a single Ψ at the first or second codon position resulted in repression of translation of the full-length ErmCL peptide by about 30%, with the strongest repression at the third codon position (Figure 2A and B). Employing the recombinant PURExpress system confirmed these results (Figures 2C and 3C and Table 1). Due to a proposed role of Ψ on rewiring of the genetic code (29), we also performed mass spectrometry analysis on the purified ErmCL peptide. However, in none of the tested constructs an alteration of the respective cognate amino acid could be identified, thus excluding the possibility of recoding by Ψ-mRNA modification (Supplementary Figure S2C).

### 2-OCH_3_

In addition to previously reported modifications in coding sequences of mRNAs, we also investigated 2′-OCH_3_, which, up till now, has not been reported to occur within bacterial or eukaryal mRNAs, respectively. Thereby, we introduced the methoxy-groups within riboses of the wt CAA codon sequence. The introduction at the first nucleotide of the CAA codon marginally reduced the translation of the full-length peptide by 10–15%, whereas the 2′-O-methyl group at the second nucleotide of the codon significantly reduced the yield by almost 90%. At the wobble position (i.e. third position of the codon) the reduction was only 50% (Figure 2A and B).

**Figure 5.**
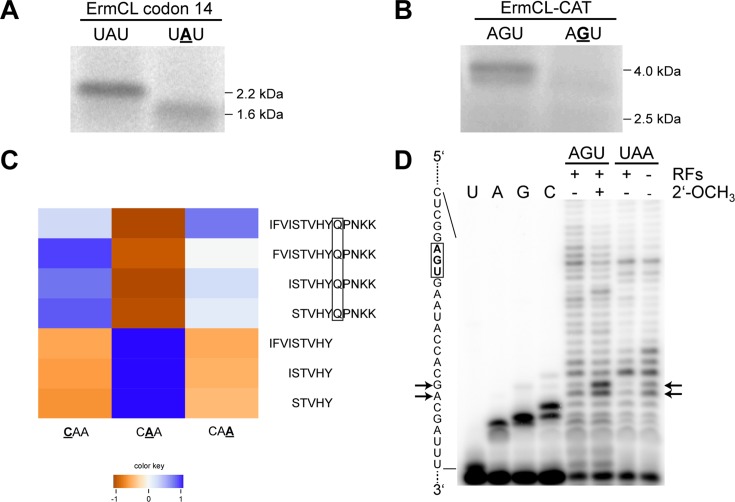
The 2′-OCH_3_ modification at the second codon nucleotide within various mRNAs leads to translation of truncated peptides (**A**) Translation product of the ErmCL peptide gene carrying the 2′-OCH_3_ modification at position 2 (bold and underlined) within the 14th codon of the peptide. The full-length product is 2.2 kDa, whereas the truncated peptide is 1.6 kDa. (**B**) A fusion mRNA of ErmCL and 15 codons of chloramphenicol acetyl transferase (CAT) was constructed. The second nucleotide at the 22nd codon of the ErmCL-CAT mRNA carries the 2′-OCH_3_ modification (bold and underlined). The full-length chimeric peptide is 4.0 kDa, whereas the truncated peptide is 2.5 kDa. (**C**) Analysis of translation products of 2′-O-methylated mRNAs using MS. mRNAs carrying a 2-OCH_3_ at the indicated codon position (bold and underlined) were translated and analyzed by MS. The frequencies of the discovered fragments are illustrated in log2 scale. The translation product of the mRNA harboring a methylated nucleotide at the second codon position did not allow the synthesis of a full-length peptide and resulted in a termination of translation prior to glutamine (boxed). (**D**) Analyses of the presence of a stalled ribosomal complex translating the 2′-OCH_3_ modified ErmCL-CAT mRNA. The sequence of the ErmCL-CAT mRNA is depicted on the left, the 22nd codon (AGU) harboring the 2′-OCH_3_ modification at the second codon position is boxed and the toeprinting sites are marked with arrows. The mRNAs were translated with the PURExpress *in vitro* translation system and the presence of the 2′-OCH_3_ at the second codon position of AGU is indicated. As a positive control for a toeprint signal, the 22nd codon of the ErmCL-CAT mRNA was mutated from AGU to the stop codon UAA and this mRNA was translated in the absence of all release factors (RFs). The sequencing lanes were obtained by primer extension in the presence of one ddNTP and the three corresponding dNTPs.

Employing the PURExpress translation system an even more pronounced inhibitory effect was observed (Figures 2C and 3D and Table 1). 2′-OCH_3_ at the second codon nucleotide almost completely abolished synthesis of the full-length peptide. Since 2′-O-methylation of the ribose at this codon position showed the strongest repression of all modifications, we introduced it at the 14th codon (UAU) of the ErmCL mRNA to exclude a codon- or a position-specific effect. In addition, an alternative mRNA sequence was applied to additionally change the sequence context (Supplementary Figure S1B). In both cases almost complete termination of translation of the full-length ErmCL peptide was observed (Figure 5A and B).

Peptide products, resulting from the translation of 2′-O-methylated mRNAs, were analyzed by MS, and did not reveal a change in the respective cognate amino acid sequence compared to the unmodified mRNAs (Supplementary Figure S2D). In addition, truncated peptides, resulting from the translation of the mRNA modified at the second codon position, did not harbor glutamine at their carboxy terminus (Figure 5C) indicating that 2′-O-methylation prevents a step prior to peptide bond formation at the modified codon.

To investigate, whether nucleotide modifications would result in termination of protein synthesis, i.e. in dissociation of the mRNA/tRNA/peptide complex from the ribosome, or in ribosomal stalling, we performed a toe-printing assay (40). Thereby, we focused on the nucleotide modification 2′-OCH_3_ at the second codon position, which resulted in significant reduction of the full-length protein, as assessed previously by our *in vitro* translation assay. As a control, we employed an mRNA with a UAA stop codon at the corresponding codon, and assayed translation of the ErmCL mRNA in the presence or absence of release factors (RFs; Figure 5D). While a moderate increase in the toeprint signal could be observed in the translation reaction lacking RFs, a significant increase could be observed employing the 2′-O-methylated mRNA, compared to the unmodified mRNA, thus indicating ribosome stalling. Thereby, the toeprint signal corresponded to a stalled ribosome complex, exhibiting the modified codon positioned at the A-site of the ribosome (Figure 5D).

## DISCUSSION

In Bacteria and Eukarya, numerous RNA modifications have previously been identified in coding (mRNAs) as well as non-coding RNAs (ncRNAs). However, their presence and function within mRNAs has only very recently been appreciated (17–18,26,51). Due to the development of high-throughput screening technologies, nucleotide modifications were shown to be a common feature within mRNAs, raising the question of their biological significance (12–16). Thereby, the function of nucleotide modifications in mRNAs was mainly attributed to altered mRNA stability and processing, altered binding affinities of proteins to the respective mRNAs (thereby influencing translation efficiency) or to altered subcellular localizations of the modified mRNAs (reviewed in 10,18,52).

To obtain a more detailed picture on the impact of mRNA modifications on translation, in particular during elongation, we aimed for a systematic approach, employing single mRNA modifications at defined positions within a codon of the bacterial ErmCL mRNA. In addition, we compared *in vitro* translation assays, employing whole cell extracts with reactions employing purified translation components (PURExpress). This enabled the direct investigation of translation by elimination of the majority of nucleases or proteases, respectively (53). In addition, the likelihood of the presence of so far unidentified proteins and factors, which might influence the stability of the introduced modifications, can be minimized (17).

By this approach, we observed that the investigated mRNA modifications inhibited translation to different extents, while inhibition of translation was also strongly dependent on the position of the modified nucleotide within the codon (Table 1). Thereby, m^6^A, recently found to be widespread within bacterial mRNAs (21), reduced the synthesis of the full-length peptide when positioned in the coding sequence of the mRNA. Notably, introducing an ‘all m^6^A codon’ (i.e. m^6^A at all three codon positions) completely terminated protein synthesis, consistent with an additive effect of nucleotide modifications (Supplementary Figure S4).

This finding is in contrast to previous studies employing eukaryal *in vitro* translation systems, which showed that the presence of m^6^A is only inhibitory when more than 5% of adenines were substituted by m^6^A (35), while a single m^6^A was reported to even stimulate translation (34). Furthermore, in our bacterial system the introduction of a single m^5^C or Ψ within the coding sequence resulted in reduced product yields. In contrast, eukaryal translation systems do not seem to be negatively affected by these modifications (35,54). Thereby, it has been shown that protein levels, resulting from Ψ-containing mRNAs, were considerably higher and these high levels were observed for a prolonged time (23,43). This effect was attributed, however, to an enhanced stability of the mRNA, rather than to a stimulation of the translation machinery.

It is also striking that the effect of modified mRNAs varied within different eukaryal *in vitro* translation systems: whereas Ψs stimulated translation in a rabbit reticulocytes system, no such stimulation could be observed using wheat germ extracts (34). Considering the central role of the translation machinery in all organisms, including its highly conserved decoding mechanism, such differences in the effects of mRNA modifications between bacterial and eukaryal translation systems are remarkable and will require further investigations (55).

In our translation assays, the strongest inhibition of protein synthesis by a nucleotide modification was observed for the 2′-O-methylation within mRNAs. Thereby, it is noteworthy that the methylated nucleotide at the second codon position almost completely abolished translation, whereas the nucleotide modification at the first codon position was largely tolerated (Figure 2). MS analysis revealed that 2′-O-methylation at the second codon position did not result in the incorporation of the corresponding amino acid (Figure 5C).

Subsequent toeprint analysis of the translation complex, containing 2′-OCH_3_ at the second codon position, was consistent with stalling of the ribosomal complex, thereby placing the modified codon in the ribosomal A-site (Figure 5D). This suggests that the corresponding glutamine-tRNA^Gln^ might be unable to bind to the A-site, as previously observed with other 2′-ribose modifications (45,46).

Toeprint analysis also indicates that at least one nucleotide modification, i.e. 2′-OCH_3_, results in stalling of the ribosomal translation complex. Interestingly, the toeprint signal appeared more pronounced, compared to an mRNA exhibiting an UAA stop codon at the modification site while lacking RFs in the translation assay. However, future investigations will have to demonstrate whether this is a more general mechanism and whether stalling of the ribosomal complex might subsequently result in dissociation of the stalled ribosomal complex.

As deduced from recent high-resolution crystal structures, the 2′-OH of the mRNA, located at the second codon position in the ribosomal A-site, points toward the 16S rRNA (56). A substitution of the hydroxyl group by a more bulky methoxy group might thus cause a steric hindrance, leading to reduced binding of the aa-tRNA (amino acyl tRNA) anticodon to the codon of the modified mRNA. In contrast, a methyl group at the first or third nucleotide of the codon appears to be less sterically restricted and therefore binding of an aa-tRNA might be feasible. As for the remaining nucleotide modifications, however, more experimental data are required to interpret the effects resulting from the incorporation of m^5^C, m^6^A or Ψ during peptide synthesis.

A long-standing enigma, considering mRNA modifications, concerns the question whether they are able to rewire the genetic code. As an example, the incorporation of Ψs in the stop codon was shown to result in efficient nonsense repression (27,28) and thus indicated that mRNA modifications are indeed able to influence the identity of the protein product. Computational calculations have supported this hypothesis (29). In contrast, recent analyses of protein products, resulting from Ψ-containing mRNAs, did not reveal amino acid alterations (15).

To shed light on this discrepancy, we analyzed all peptides resulting from translation of modified mRNAs by MS (Supplementary Figures S2 and S3). Thereby, we observed that the presence of Ψ, m^6^A or 2′-O-methylation of riboses did not change the amino acid identity of the respective codon at any codon position investigated. In contrast, positioning m^5^C at the second codon position resulted in an incorporation of a small amount of leucine (4%) instead of proline (Figure 4 and Supplementary Figure S5). Thereby, the introduction of m^5^C appears to enable formation of a G-U wobble base pair at the second codon position, thus allowing the near cognate aa-tRNA (i.e. leucine tRNA) to bind to the ribosome. However, further investigations are required to mechanistically elucidate how the m^5^C modification might alter the base pairing characteristics and whether the amino acid exchange is able to alter protein function or activity. Nevertheless, to our knowledge this is the first experimental evidence of a rewiring of the genetic code by m^5^C.

In times of a constantly expanding RNA modification repertoire a direct impact of mRNA modifications on ribosomal translation is becoming increasingly plausible. Currently, a rapidly growing number of organisms is identified to harbor modified mRNA coding sequences. In this work, we systematically investigated the impact of single nucleotide modifications on the translation machinery. Our results strongly suggest that in Bacteria, modifications within coding sequences of mRNAs might be employed as regulators of ribosomal translation. Thereby, depending on the type and the codon position of the modification, protein synthesis can either be prematurely terminated or novel proteins might be generated containing an altered amino acid composition. Premature translation termination will result in decreased protein levels of the full-length product.

It remains to be elucidated, however, whether protein fragments, generated by premature translation termination, or proteins containing altered amino acids (see above), indeed fulfill biological roles. Translation modulation by mRNA modifications is thus reminiscent of ‘transcriptional rewiring’, i.e. RNA editing, which allows synthesis of an edited and thus altered protein variant, in addition to the canonical protein. Future studies will have to investigate function, if any, of prematurely terminated proteins as well as elucidate mechanisms to introduce these site-specific modifications within mRNAs.

In this study, we have focused predominantly on single site modifications within a codon of an mRNA, in order to reduce the complexity of the investigated translation system. However, by incorporation of several modified nucleotides, e.g. within one codon or several codons, is likely to result in an additive effect. This assumption is corroborated by the introduction of three consecutive m^6^A or deoxy-nucleotides within one codon of the ErmCL mRNA, which completely abolished translation, while single nucleotide substitutions exhibited weaker effects. In conclusion our results strongly suggest that mRNA modifications are employed as important tools of translation regulation of gene expression.

## Supplementary Material

SUPPLEMENTARY DATA
