# Supplementary material for: Nucleotide modifications within bacterial messenger RNAs regulate their translation and are able to rewire the genetic code
Source: Nucleic Acids Res. 2015 Nov 17;44(2):852–62. doi: 10.1093/nar/gkv1182 (PMC4737146; doi:10.1093/nar/gkv1182)
Supplement: SUPPLEMENTARY DATA [file supp_44_2_852__index.html]

Nucleotide modifications within bacterial messenger RNAs regulate their translation and are able to rewire the genetic code — SUPPLEMENTARY DATA 

# Nucleotide modifications within bacterial messenger RNAs regulate their translation and are able to rewire the genetic code

## SUPPLEMENTARY DATA

- SUPPLEMENTARY DATA
