# Supplementary material for: Nucleotide modifications within bacterial messenger RNAs regulate their translation and are able to rewire the genetic code
Source: Nucleic Acids Res. 2015 Nov 17;44(2):852–62. doi: 10.1093/nar/gkv1182 (PMC4737146; doi:10.1093/nar/gkv1182)
Supplement: SUPPLEMENTARY DATA [file supp_gkv1182_Supporting_DATA_resubmission_UPLOAD_III.docx]

**SUPPLEMENTARY DATA**

**Material and Methods**

**Mass spectrometry of the synthetic RNA containing m^5^C at the second codon position (TH133)**

Mass spectrometry (MS) experiments were performed on a 7 Tesla Fourier transform ion cyclotron resonance (FT-ICR) instrument equipped with an electrospray ionization (ESI) source (Bruker). For ESI, RNA was desalted using Vivaspin 500 PES centrifugal concentrators (MWCO 3,000) (Sartorius) as described previously (57), and a 1 µM solution of TH133 RNA in 1:1 H_2_O/CH_3_OH with piperidine and imidazole as additives (25 mM each) was prepared. Using polyethylene glycol 1000 as internal calibrant, a mass value of 7367.075 Da was determined for the most abundant isotopic peak of TH133 RNA (theoretical mass: 7367.068 Da). Collisionally activated dissociation (CAD) of (M-nH)^n-^ ions of TH133 RNA produced characteristic ***c***- and ***y***-type fragment ions from phosphodiester backbone cleavage (58) whose mass values confirmed the 23 nt sequence with methylation at residue 7 (Figure S6A). In a two-stage CAD experiment, fragment ions comprising the first 7 residues were isolated and fragmented, which again produced ***c***- and ***y***-type fragments (Figure S6B) whose calculated monoisotopic mass values were used for internal calibration (standard deviation 0.2 ppm). Experimental and calculated MS signals for the ***y***-type dinucleotide fragment ions comprising cytosine and 5-methylcytosine, C-m^5^C (Figure S6C) are illustrated in Figure S6D (upper and middle trace). No evidence was found for the presence of RNA in which hydrolytic deamination or artifacts during solid phase synthesis caused conversion of 5-methylcytosine into 5-methyluracil, as no signals from ***y***-type dinucleotide fragment ions C-m^5^U (Figure S6E) were observed (Figure S6D, upper and lower traces). Analysis of the signal-to-noise ratio at m/z 623.0911 gave an upper limit for the potential presence of 5-methyluracil in TH133 RNA of 1.7%.

57. Breuker, K. (2014) Characterization of ribonucleic acids and their modifications by Fourier transform ion cyclotron resonance mass spectrometry. *Nucleic Acids in the Gas Phase*, ed Gabelica V (Springer, Heidelberg), 185-202.

58. Riml, C., Glasner, H,, Rodgers, M. T., Micura, R., Breuker, K. (2015) On the mechanism of RNA phosphodiester backbone cleavage in the absence of solvent. *Nucleic Acids Res* **43**, 5171-5181.


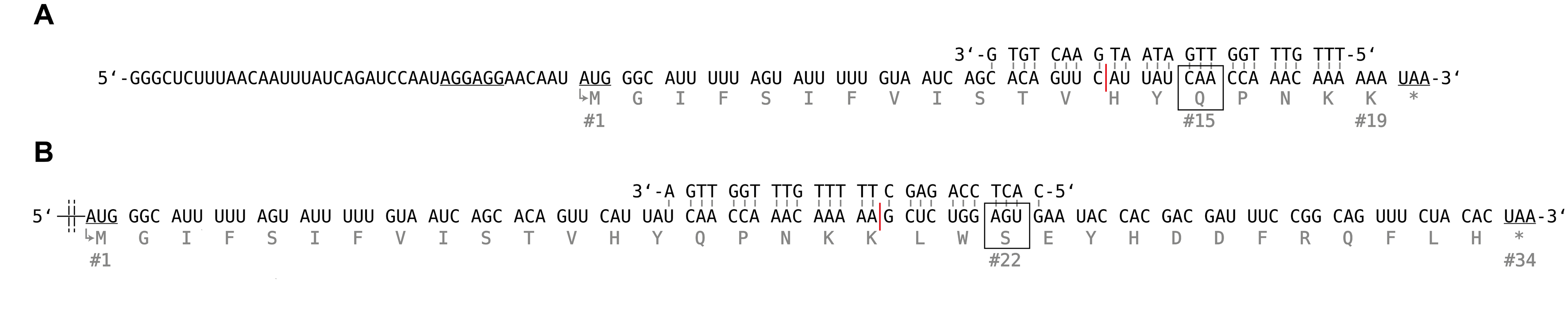


**Figure S1:** The mRNAs carrying a site-specific modification were generated via a splinted ligation approach. The 5’- part of the mRNA was *in vitro* transcribed, the 3’-part harboring the modification (modified codon is framed) was chemically synthesized. A complementary bridging DNA-oligonucleotide (splinter) was used to correctly position the mRNA halves during ligation. The ligation site is depicted as a red line. Start and stop codons are underlined. (**A**) Nucleotide sequence of the ErmCL peptide gene. The Shine-Dalgarno sequence is underlined. ErmCL codes for 19 amino acids (amino acid sequence depicted in grey), the site-specific modification was introduced within the 15^th^ codon at the first, second, or third nucleotide, respectively. To investigate all three positions of a codon individually, the corresponding wild type sequence was changed from CAA to AAA for m^6^A, CCC for m^5^C, UUU for Ψ, respectively. (**B**) A fusion mRNA of ErmCL and 15 codons of the chloramphenicol acetyl transferase (CAT) was constructed. The 2’-methoxy modification was introduced at the 22^nd^ codon.


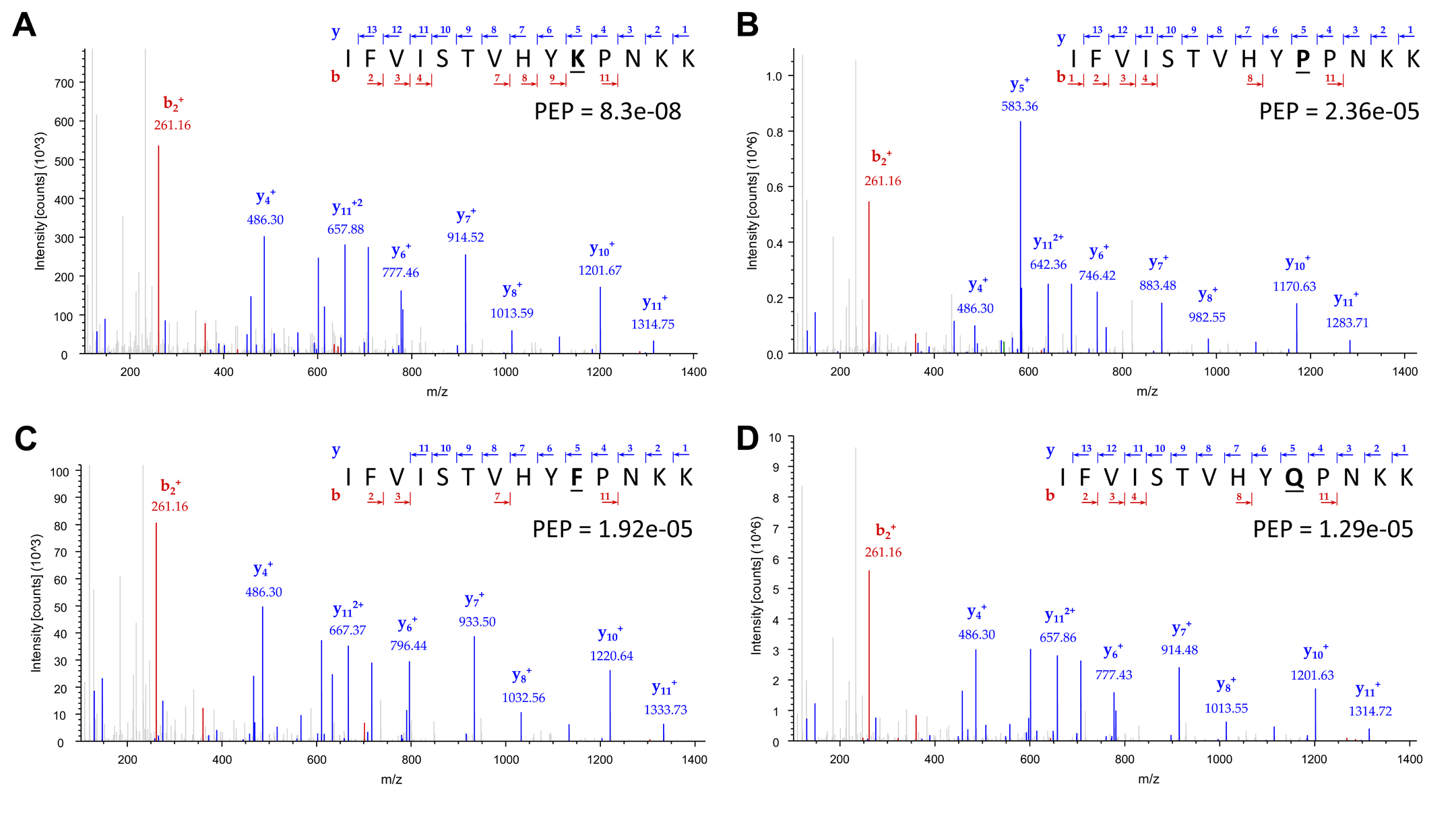


**Figure S2:** MS/MS spectra of ErmCL peptides encoded by mRNAs harboring modifications at the 15^th^ codon. The following modifications were investigated: (**A**) m^6^A at the codon AAA encoding the amino acid lysine K; (**B**) m^5^C at the codon CCC encoding proline P; (**C**) Ψ at the codon UUU encoding phenylalanine F; and (**D**) 2’-OCH_3_ on position 1 and 3 of the codon CAA that encodes glutamine Q. The altered mRNA sequence results in peptides differing in their amino acid sequence (bold and underlined amino acid), which can be observed in a mass shift of the y-ion fragment series starting at the y5 fragment. The posterior error probability (PEP) indicates the probability that this annotation is incorrect.


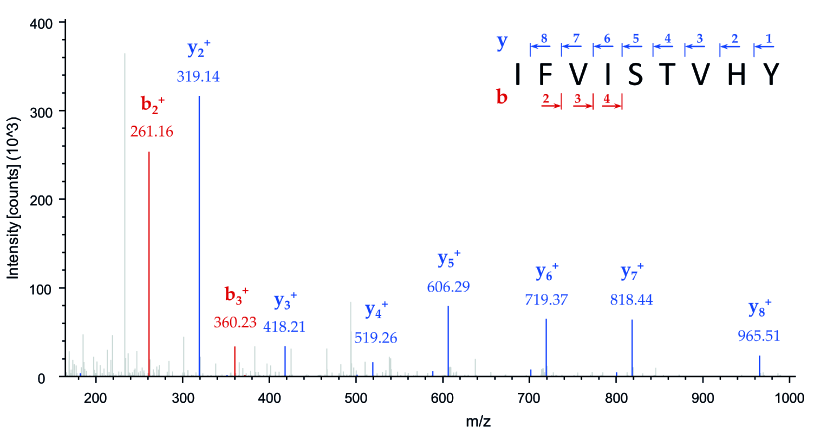


**Figure S3**: MS/MS spectrum of the ErmCL peptide encoded by the mRNA harboring a 2’-OCH_3_ modification at the second nucleotide of the 15^th^ codon. Annotated peptide fragments are shown in blue (y-ions) and red (b-ions), according to the general nomenclature of fragment ions. The posterior error probability (PEP) indicates the probability that this annotation is incorrect.


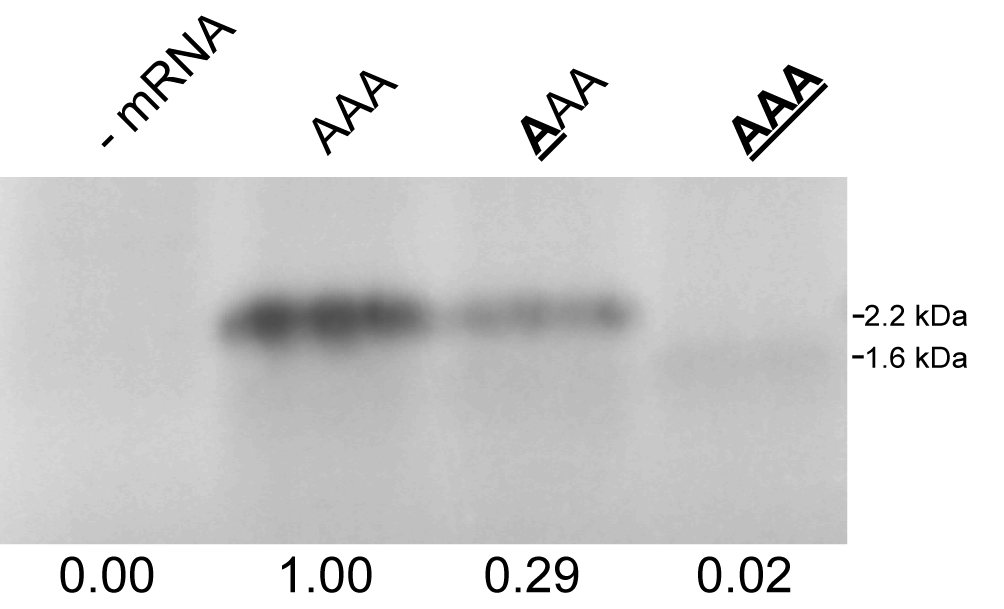


**Figure S4:** *In vitro* translation of ErmCL mRNAs carrying m^6^A at the indicated positions (bold and underlined). Quantification of *in vitro* translated mRNAs employing the PURExpress system is depicted below. The product yield of the translation of the unmodified mRNA was set to one.


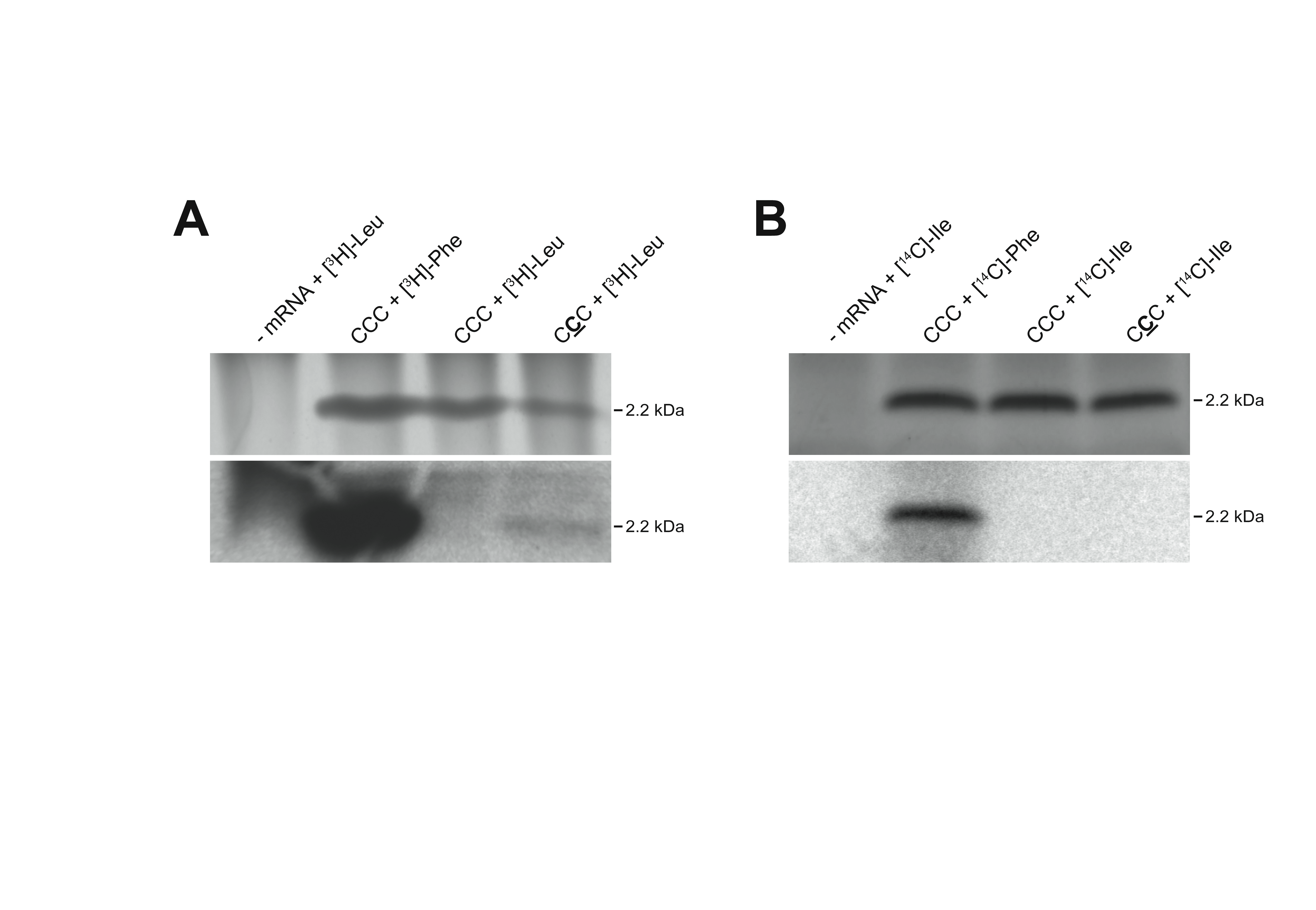


**Figure S5**: Incorporation of leucine/isoleucine into ErmCL peptides. No mRNA control, the unmodified ErmCL mRNA (CCC) and the mRNA containing m^5^C at the second codon position (C**C**C) were translated in the presence of [^3^H]-labeled leucine (**A**) or [^14^C]-labeled isoleucine (**B**). The ErmCL mRNA does not contain a leucine-codon and all isoleucine codons were mutated to methionine codons. As a size marker the unmodified ErmCL mRNA was translated in the presence of [^3^H]- or [^14^C]-labeled phenylalanine. The full-length product is 2.2 kDa. The upper panel shows the silver-stained protein gel, whereas in the lower panel the corresponding autoradiography is shown.


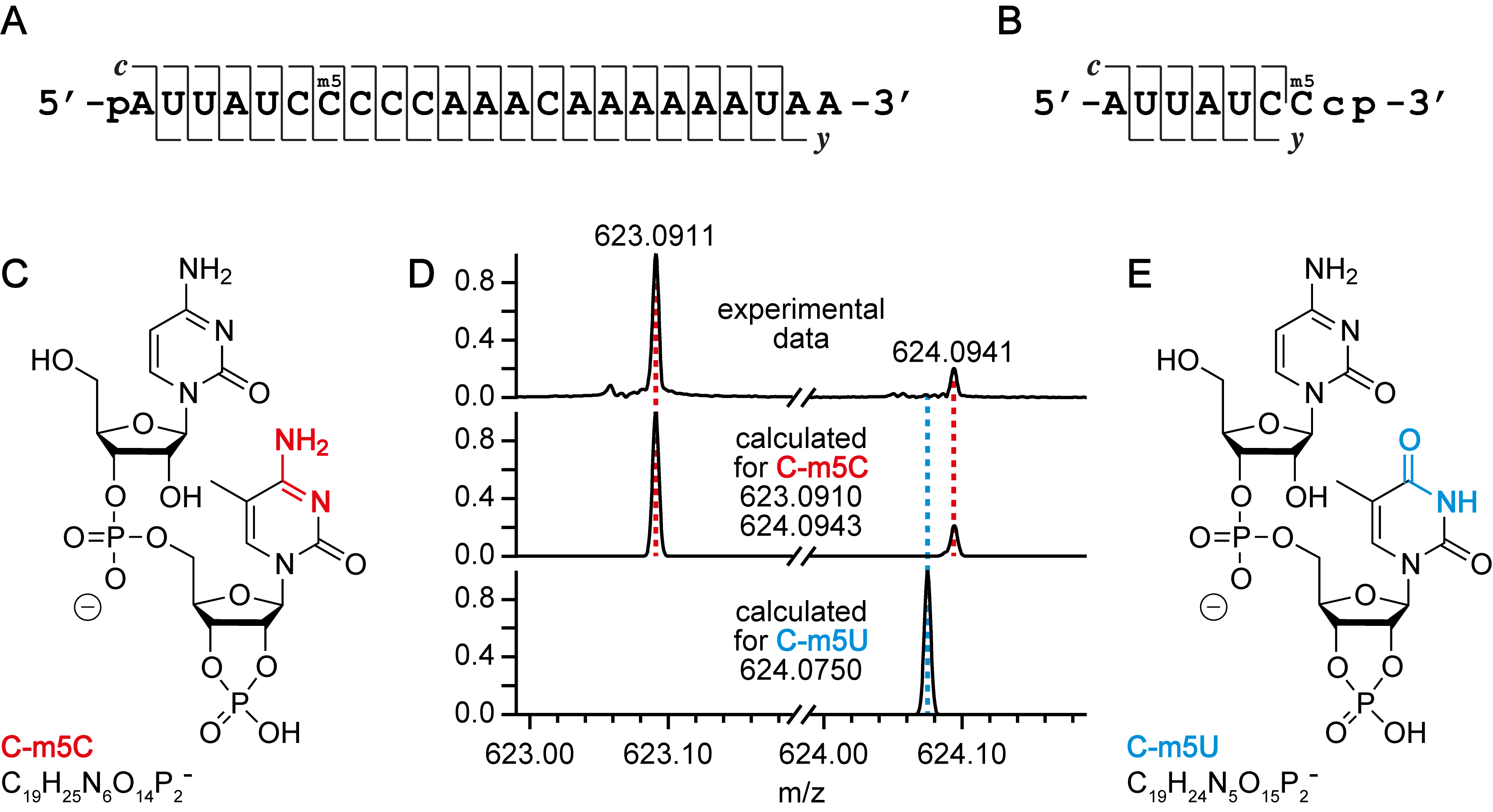


**Figure S6:** Characterization of TH133 (synthetic RNA oligonucleotide containing m^5^C at the second codon position) by MS. CAD fragment ion maps for TH133 (**A**) and its fragment comprising the first 7 residues (**B**), which was further dissociated to produce the dinucleotide fragment ions comprising cytosine and 5-methylcytosine, C-m^5^C (**C**). MS signals for C-m^5^C were observed experimentally (**D**) but none were found for C-m^5^U (**E**).
